# Influence of social determinants of health in family effectiveness*

**DOI:** 10.15649/cuidarte.2805

**Published:** 2023-05-29

**Authors:** Fernanda Lise, Eda Schwartz, Lílian Moura De-Lima-Spagnolo, Jeanne Marie Stacciarini

**Affiliations:** 1 . Universidade Federal de Pelotas, Pelotas, Brazil. E-mail: fernandalise@gmail.com Universidade Federal de Pelotas Universidade Federal de Pelotas Pelotas Brazil fernandalise@gmail.com; 2 . Universidade Federal de Pelotas, Pelotas, Brazil. E-mail: edaschwa@gmail.com Universidade Federal de Pelotas Universidade Federal de Pelotas Pelotas Brazil edaschwa@gmail.com; 3 . Universidade Federal de Pelotas, Pelotas, Brazil. E-mail: lima.lilian@gmail.com Universidade Federal de Pelotas Universidade Federal de Pelotas Pelotas Brazil lima.lilian@gmail.com; 4 . University of Florida, Gainesville, United States. E-mail: jeannems@ufl.edu University of Florida University of Florida Gainesville USA jeannems@ufl.edu

**Keywords:** Family nursing, Nursing Evaluation, Self-efficacy, Enfermería de la Familia, Evaluación de Enfermería, Autoeficacia, Enfermagem familiar, Avaliagáo de Enfermagem, Autoeficácia

## Abstract

**Introduction:**

The Social Determinants of Health are defined by the living conditions of the population, how they are born, grow, live, work and age, and are considered as the main responsible for the health of populations.

**Objective:**

To evaluate the influence of social determinants of health on effectiveness of family functioning.

**Materials and Methods:**

A quantitative study was carried out with 200 participants between 2017 and 2018. To assess the correlation between proximal, intermediate and distal social determinants of health and the level of effectiveness of family functioning, home visits were made to 100 families who responded the Assessment of strategies in Family Effectiveness instrument and a sociodemographic questionnaire. Data were analyzed by simple frequency, Spearman's correlation coefficient (p) (p <0.05), and subjected to descriptive analysis from the perspective of systemic organization.

**Results:**

The social determinants that showed a positive correlation coefficient with the level of effectiveness of family functioning were the years of study and the fixed family income. There was a negative correlation with the main source of income (number of occupations) or source of income and the presence of other members or relatives in the family.

**Discussion:**

Investments in education can improve the family's ability to organize, solve or prevent adverse events, increase income and provide more time for family relationships and the achievement of congruence, as evidenced by other authors.

**Conclusions:**

The proximal and intermediate social determinants of health influenced the level of effectiveness of family functioning.

## Resumen

**Introducción:**

Los Determinantes Sociales de la Salud están definidos por las condiciones de vida de la población, cómo nacen, crecen, viven, trabajan y envejecen, y son considerados como los principales responsables de la salud de las poblaciones.

**Objetivo:**

Evaluar la influencia de los determinantes sociales de la salud en la efectividad del funcionamiento familiar.

**Materiales y Métodos:**

Se realizó un estudio cuantitativo con 200 participantes entre 2017 y 2018. Para evaluar la correlación entre los determinantes sociales de salud proximales, intermedios y distales y el nivel de efectividad del funcionamiento familiar, se realizaron visitas domiciliarias a 100 familias que respondieron el instrumento Evaluación de estrategias en Efectividad Familiar y un cuestionario sociodemográfico. Los datos fueron analizados por frecuencia simple, coeficiente de correlación de Spearman ( p ) (p <0,05 ), y sometidos a análisis descriptivo desde la perspectiva de la organización sistémica.

**Resultados:**

Los determinantes sociales que mostraron un coeficiente de correlación positivo con el nivel de efectividad del funcionamiento familiar fueron los años de estudio y el ingreso familiar fijo. Hubo una correlación negativa con la principal fuente de ingresos (número de ocupaciones) o fuente de ingresos y la presencia de otros miembros o familiares en la familia.

**Discusión:**

Las inversiones en educación pueden mejorar la capacidad de la familia para organizarse, solucionar o prevenir eventos adversos, aumentar los ingresos y brindar más tiempo para las relaciones familiares y el logro de la congruencia, como lo evidencian otros autores.

**Conclusiones:**

Los determinantes sociales proximales e intermedios de la salud influyeron en el nivel de efectividad del funcionamiento familiar.

## Resumo

**Introdugáo:**

Os Determinantes Sociais da Saúde sao definidos pelas condigóes de vida da populagáo, como nascem, crescem, vivem, trabalham e envelhecem, sendo considerados como os principais responsáveis pela saúde das populagóes.

**Objetivo:**

Avaliar a influencia dos determinantes sociais da saúde na eficácia do funcionamento familiar.

**Materiais e Métodos:**

Foi realizado um estudo quantitativo com 200 participantes entre 2017 e 2018. Para avaliar a correlagáo entre os determinantes sociais proximais, intermediários e distais da saúde e o nível de eficácia do funcionamento familiar, foram realizadas visitas domiciliares a 100 famílias que responderam o instrumento Avaliagáo de estratégias em eficácia familiar e um questionário sociodemográfico. Os dados foram analisados por frequencia simples, coeficiente de correlagáo de Spearman ( p ) (p < 0,05 ), e submetidos a análise descritiva sob a ótica da organizagáo sistemica.

**Resultados:**

Os determinantes sociais que apresentaram um coeficiente de correlagáo positiva com o nível de efetividade do funcionamento familiar foram os anos de estudo e a renda familiar fixa. Houve correlagáo negativa com a principal fonte de renda (número de ocupagóes) ou fonte de renda e a presenga de outros membros ou parentes na familia.

**Discussao:**

Investimentos em educagáo podem melhorar a capacidade da família de se organizar, resolver ou prevenir eventos adversos, aumentar a renda e proporcionar mais tempo para as relagóes familiares e para o alcance da congruencia, conforme evidenciado por outros autores.

**Condusdes:**

Os determinantes sociais proximais e intermediários da saúde influenciaram o nível de efetividade do funcionamento familiar.

## Introduction

Social Determinants of Health can be defined by the living conditions of the population, how they are born, grow, live, work and age. These circumstances are shaped by the distribution of money, power and resources at the global, national and local levels^1^. In this context, social and environmental factors are the main responsible for the health of the populations^2^.

Thus, the effectiveness of family is the result of a set of situations and conditions, which encompass the individual and the environment, since the assessment of functionality concerns quality, which indicates how a particular function is developed. Therefore, functionality is a component of health, considering the environment as a facilitator or as a barrier to the performance of activities^3^.

The strategies used by families to function effectively can be assessed using instruments^4^. In this study, the Assessment Strategies in Family-Effectiveness ASF-E was chosen based on the theoretical framework of systemic organization, which aims to achieve gols for maintaining family health, for which, the family is organized to function as a system and respond to the requirements of each member^5^. In addition, scientific evidence proves its validity in assessing the level of health or the effectiveness of family functioning^6^^-^^10^. This instrument was adapted and validated to be used with Brazilian families, ASF-E/Brazil^11^. Based on these considerations, this study sought to identify the correlation of sociodemographic variables (gender, age, education, marital status, children, number of family members, occurrence of chronic diseases and family income) related to social determinants of health, with the effectiveness in family functioning. Once knowing how the social determinants of health influence the health of the family, supported by the systemic organization, with the use of the ASF-E, can contribute to the evaluation and nursing intervention with the families, and promote the improvement on the families health. This study aimed to assess the influence of social determinants of health on family-effectiveness functioning.

## Material and Methods

This was an analytical and cross-sectional study. Data were collected during home visits to 100 families living in the area covered by four Basic Health Units with Strategy in Health Family in the municipality of Pelotas, State of Rio Grande do Sul, Brazil, between 2017 and 2018.

The sample consisted of 200 people, two from each of the 100 families selected by non-probabilistic convenience sampling^12^. Criteria for inclusion in the study were age over 18 years, knowing how to read in Portuguese, the possibility of participation of two members of the same family and belonging to the area covered by the Health Units, in the city of Pelotas, State of Rio Grande do Sul, Brazil. The only criterion for exclusion was not being able to communicate verbally to answer the questions. Among the families approached, around 10.0% did not meet the inclusion criterion “possibility of participation of two family members”.

The approach to families occurred during a home visit by the researcher accompanied by community health agents and/or a nursing undergraduate student. On this occasion, the objectives of the study, the two instruments and the Informed Consent Form (ICF) were presented. After accepting to participate, the ICF was read and signed in two copies, one for the researcher and the other for each participant. The average duration of the visit was one hour and for families to complete the ASF-E/Brazil instrument, 20 minutes. Some visits were made after business hours to meet the needs of the family who were at work.

Two instruments were used for data collection, the first was a sociodemographic questionnaire, developed by the researcher, based on the Social Determinants of Health model^13^. Such variables related to socioeconomic, demographic and health conditions were considered as proximal determinants: sex, age, occurrence of chronic diseases. The intermediate determinants were: marital status, presence of children, number of family members, members coexisting with the family, other members or relatives; distal determinants: education, origin of the main source of family income, income, number of people contributing to income and number of people depending on income.

The second instrument, was the ASF-E/Brazil^11^, which assesses family health or effectiveness-family functioning, is a nominal scale, self-administered and has 20 items, which measure family processes. Each item has three alternatives scoring 1, 2 or 3 points. The effectiveness of family functioning is considered high when the alternative chosen is #3, medium to alternative #2 and low to alternative #1. As for example, the options in item 1 of the instrument and their respective options: There is anger or sadness in our family #1; People in our family do not openly express their feelings #2; Our family is happy, in general #3. The total value of the instrument is 60 points; results between 48 and 60 points are considered high effectiveness of family functioning, 34 - 47 intermediate and 20 - 33 points, low.

Data were organized in an Excel for Windows® spreadsheet and analyzed using a statistical software R. For each variable analyzed, the value observed in the sample (n) and the percentage (%) were expressed. The Spearman correlation coefficient (p) with significance of p <0.05 evaluated the relationships involving the level of effectiveness of family functioning and the sociodemographic and health variables were evaluated. The following classification of the correlation coefficients was adopted: <0.4 weak correlation, > 0.4 to <0.5 moderate correlation, and > 0.5 strong correlation^14^. The validated information was exported to the statistical package Stata/MP version 14.0. for data processing. The database was stored in Mendeley Data15.

This study was approved by the Research Ethics Committee #2.088.36. The ethical precepts of Resolution 466/12 of the National Health Council, which regulates research involving human beings^16^, were respected.

## Results

Results of sociodemographic and health variables were presented according to the classification of social determinants of health into proximal (Table 1), intermediate (Table 2) and distal (Table 3) determinants. Of the total of 200 participants, 65.0% were female, aged between 18 and 50 years old (50.0%) and reported the occurrence of chronic diseases among family members (66.0%) (Table 1).

Regarding marital status, 61.0% participants were married or living in a stable relationship. The presence of children in the households was 80.0% and the predominant number of members in the families was four to six in more than half of the sample. When asked about the members coexisting with the family, there was a predominance of father, mother and children in 52.0% families and the age of the family members was mostly under 9 years old with 41.5% (Table 2).

**Table 1:** Proximal determinants of health of the participants of the 100 families (N = 200). Pelotas, State of Rio Grande do Sul, Brazil, 2020.

	Variable	(%)
Sex		
Female		65.0 (130)
Male		35.0 (70)
Age		
18 a 60		70.5 (141)
> 61		29.5 (59)
Chronic disease		
None		43.5 (87)
AH		37.5 (75)
DM		2.5 (05)
AH + DM		16.5 (33)

*AH- Arterial Hypertension; DM - Diabetes Mellitus*

**Table 2:** Intermediate determinants of health of the participants of the 100 families (N = 200). Pelotas, State of Rio Grande do Sul, Brazil, 2020.

Variable	(%)
Marital Status	61.0(122)
Married or in a stable relationship	22.5(45)
Single	16.5(33)
Divorced; separated or widowed	
Children	
No	19.5(39)
Yes	80.5(161)
Number of family members	
2 to 3	46.0(92)
4 to 6	50.5(101)
>7	3.5(07)
Members coexisting with the family	52.0(104)
Father, mother and child(ren)	37.0(74)
Father, mother, child(ren), relatives and	11.0(22)
other members	
Mother and child(ren)	
Age of the family members	
<9 years	41.5(83)
10 to 21	21.5(43)
>21	37.0(74)

As for the years of study, there was a predominance of people with up to nine years of schooling, 48.0%. As for the main source of income, employment predominated in 35.5%, and retirement by age/ years of work stood out with 27.5% participants. When asked about the value of fixed family income, 35.5% reported income above three minimum wages. As for the number of people who contribute to the production of fixed family income, 49.0% mentioned two people, while three dependents on fixed family income predominated in 35.0% (Table 3).

**Table 3:** Distal determinants of health of the participants of the 100 families (N = 200). Pelotas, State of Rio Grande do Sul, Brazil, 2020.

Variable	(%)
Years of study	
Did not study	2.0(04)
5 to 9 (Elementary School)	48.0(86)
10 to 12 (High School)	26.0(52)
15 to 16 (Higher Education)	24.0(48)
Main source of income	
Employment	35.5(71)
Family income	19.0(38)
Benefit (pension or sickness benefit)	17.5(35)
Retirement by age/years of work/disa- bility	27.5(55)
Other1/	0.5(01)
Fixed family income2/ In minimum wage	
< 1	11.5(23)
Between 1 and 2	32.0(64)
Between 2 and 3	21.0(42)
> 3	35.5(71)
Contributor in fixed family income	
1	19.0(38)
2	49.0(98)
3 to 6	32.0(63)
Dependents of fixed family income	
2	14.0(28)
3	35.0(70)
4	27.0(54)
5 to 11	24.0(48)

*1/ Other: informal work. 2/ One national minimum wage: R$ 937.00 in 2017/18.*

In the evaluation with the ASF-E/Brazil instrument, it was identified that 82.0% families had high effectiveness of family functioning.

Table 4 shows a positive correlation between the effectiveness of family functioning with the years of study (0.181 and p=0.010), proving that the more years of study, the greater the level of effectiveness of family functioning. Positive correlations were also detected with the fixed family income (0.221 and p=0.002), demonstrating the importance of work with fixed monthly payment, due to the possibility of organizing the family finances, which can bring safety and improve the result of family functionality. As with the number of family members (0.155 and p=0.028), which proves that the concentration of families with two to six members resulted in better family functioning.

**Table 4:** Correlation between the effectiveness of family functioning and the social determinants of health. Pelotas, State of Rio Grande do Sul, Brazil, 2020.

Variable	(p)	p-value
Sex	0.06	0.358
Age	0.04	0.505
Marital status	-0.09	0.161
Chronic disease	0.04	0.498
Children	-0.14	0.060
Number of family members	0.15*	0.028*
Presence of relatives or other members coexisting with the family	-0.22**	0.001**
Years of study	0.18*	0.010*
Main source of income (Number of occupations)	-0.19**	0.007**
Fixed family income	0.22*	0.002*
People who contribute to the fixed family income	0.09	0.178
People who depend on the fixed family income	0.13	0.064

Spermann correlation coefficient (p);significance (p<0.05); *Positive Correlation; **Negative correlation.

There were negative correlations between the effectiveness of family functioning and the main source of income (-0.191 and p=0.007), which means that the greater concentration of families with employment, as the main source of income, resulted in lower effectiveness of family functioning. There was a negative correlation between the effectiveness of family functioning and the presence of relatives or other members coexisting with the family (-0.229 and p= 0.001), that is, the greater the presence of other members or relatives such as mother-in-law, grandson, son-in-law, brother-in-law, nephew, uncles, cousins, the lower the effectiveness of family functioning of these families.

The lack of correlations between the effectiveness of family functioning and sex, age of the participant, chronic disease, marital status, presence of children, people who contribute to fixed family income, people who depend on fixed family income, demonstrated that these characteristics did not contribute for the functioning of the assessed families.

## Discussion

The social determinants of health influence the level of effectiveness of family functioning, especially education, fixed income, the number of members of the family group, the presence of relatives and the number of sources of income or occupations. These results may be related to the characteristics of the relationships of individuals with the environment, the exposed social conditions, as well as with the various systems that make up the family system.

Due to the socio-cultural aspects and gender construction in Brazil, the characteristics of the study population, mostly female, in which household and childcare tasks are apprehended and said to be female responsibilities^17^. Despite the social changes that have occurred in recent decades, women face challenges related to gender equality and situations of violence^18^.

The productive force and the capacity to meet the demands of the system, identified in the age range of the participants, in general, young adults, corroborates national data^19^. In Brazil, the majority of unemployed people are femal^19^, and even after women have assumed new roles in society^20^, they are exposed to situations of occupational segregation, with higher professional qualification and lower pay, when inserted in the labor market^21^^-^^24^. Added to this is the burden of double or triple shifts. The answer to these challenges can come with the realization of gender equality, one of the goals of the millennium so that women have the same opportunities and participate in decision-making^18^.

As a reflection of the reality of Brazilian families, the participants declare themselves married or in a stable relationship, as the data from the national survey pointed to the increase in informal unions. The so-called consensual union was the only one that grew in the decade, from 28.6% to 36.4%. The proportion of people married in civil and religious terms, in the same period, dropped from 49.4% to 42.9% in the decade^25^. In Brazil, a stable union was equated with marriage in the 1988 constitution, with the aim of preserving the family, and the rights and duties are very similar to that of marriage. In this relationship, their maintenance is linked to the affection and real interest between the couple and both agree to end the relationship when it becomes unsatisfactory^20^^-^^23^.

Likewise, the most common family arrangement was that of a family with children, characterized as nuclear. This data was similar to the national data of couples with children, according to the Brazilian Institute of Research and Statistics, of the total of 27.4 million couples with children, 83% live with children conceived by the couple, one sixth (16.3%) lives with stepchildren, in addition to children, or only with stepchildren (reconstituted families)^26^. In this study, in addition to the presence of children, it was identified that other members and relatives make up the family of 37.0% of the participants.

The negative correlation between the effectiveness of family functioning and the presence of other members and relatives suggests that families with the presence of in-laws, uncles, nephews, brothers, brothers-in-law, sons-in-law, grandparents and great-grandparents, tend to have a lower effectiveness in family functioning. Such condition may be related to disrespect for individuality, a stress triggering factor in extended families (in this study, without biological and reconstituted ties), given the frequent interactions. In this form of organization, with other relatives living together, the social support factor can be considered, as observed in home visits, a high number of children, which indicates the dependence on care by its members. However, in general, this form of organization may be due to economic needs in which families are organized to meet the needs of work and income^27^.

The high number of family members with some chronic disease, especially arterial hypertension and/ or diabetes mellitus, can indirectly impair family functioning. The other chronic diseases mentioned by the participants were diseases of the respiratory, renal and immune systems. This occurrence may be related to the increase in chronic non-communicable diseases (CNCD).

We highlight the importance of investments in access to formal education to improve the health level of families, given the positive correlation between years of study and the effectiveness of family functioning. This result shows the influence of the increase in years of study on the best levels of family health, as education is a transforming factor for individuals and society^18^, with the potential to improve income and the ability to solve or prevent adverse events and the extent of family congruence (harmony) or balance in family relationship^9^. Most of the participants (48.0%) declared to have studied up to 9 years, which corresponds, in Brazil, to complete elementary school^28^. Followed by 15 to 16 years of study (24.0%), which corresponds to the complete higher education level, similar to national data^28^.

Likewise, the intermediate social determinant of health, fixed income, had a positive correlation with the effectiveness of family functioning. Participants declared family income above three minimum wages (R$ 2,811.00), each minimum wage corresponded to R$ 937.00 in the period of data collection. The participants declared the contribution of two people to 49.0% and dependence on the income of three people in 35.0% families. This shows that the family income was higher than the per capita household income of the State of Rio Grande do Sul (R$ 1,635.00) and more than twice that of Brazil (R$ 1,268.00) in the period^28^.

The negative correlation between the main source of income, arising from employment and or retirement with the effectiveness of family functioning, allows to infer that the more work or pensions as the main source of income, the lower the effectiveness of family functioning. Families whose adults, in general, with children, dedicate many hours to work, consequently, have less time available for family relationships, which implies a lower family functioning, as it destabilizes the balance of systems^6^. This leads to a reflection on the social construction where men and women are exposed to work demands to satisfy the family's economic needs, which affect the family's health. In the same way, the increase in the number of retirement pensions as the main source of family income, in this study 45.0% of the income originated from pension, sickness pension or retirement by age, years of work and disability, may represent less functional capacity of members. Some participants reported having returned to work after retirement to supplement the family's income. Such a condition may require the family to organize care and/or cope with changes in routine that directly imply family relationships^29^^,^^30^.

The classification of the level of effectiveness of family functioning showed that 82.0% was at the high level. In this way, it is possible to affirm that families use strategies of effectiveness of family functioning to be healthy. This result can be related to clear limits, openness to relationships with subsystems and respect for individuality^5^. A similar result was found with Colombian families^10^.

However, in the present study, 17.0% families were classified at the intermediate level of the effectiveness of family functioning, which may be related to the families' inability to meet the demands of the subsystems. Especially the factors that decrease the level of effectiveness of family functioning, identified in this study, such as lower level of education, absence of fixed income, increase in the number of the main source of income and the presence of other members or relatives in the family dynamics. These factors can trigger tensions in the family environment and feelings of stress, which lead to the deterioration of the system, as they reduce the families' ability to organize themselves to maintain congruence.

The limitations of this study are related to the characteristics of the participants, since they were all adults and lived in an urban area. Thus, it is suggested to conduct other studies with rural families, adolescents and the elderly, healthy and/or in chronic health conditions.

Contributions to nursing practice are related to the understanding of the influence of social determinants of health, especially related to the importance of increasing years of study, income, and the number of family members in the level of effectiveness of family functioning. Nevertheless, the more work or pensions and the greater number of other members and/or relatives, the worse the family functioning, as these factors can cause tensions and stress that, according to the systemic organization, are responsible for the deterioration of the system^5^. As nurses, there is an ethical commitment to contribute to improving the health of families, carrying out interventions that modify the patterns of dysfunctional interaction, reaffirming the relevant role at all levels in practice, research and education, with the responsibility to promote health care that improves care systems and efforts to achieve equity in health^31^.

## Conclusions

The results identified the correlation of proximal (years of study, income and number of members) and intermediate (main source of income and presence of other members or relatives) social determinants of health with the effectiveness of family functioning. This allows to state that, in families with the highest level of functionality, there are the best levels of education, fixed income and are made up of two to six members. However, factors such as the presence of other members and/or relatives (such as grandparents, great-grandchildren, grandchildren, brothers-in-law, uncles and nephews) and the increase in the number of the main source of income (more than one employment or retirement in the family) erode the family system, because they are factors that trigger tensions and stress and, consequently, reduce the effectiveness of family functioning.

As the families showed a high level of effectiveness of functioning, it is possible to state that they find congruence and handle situations imposed on the system with greater skill, when the environment provides access to the necessary resources. Even so, the need to invest in political and economic efforts to provide greater access to education and work is emphasized, which contribute to improving the effectiveness of family functioning and, consequently, the level of health of families.
